# Arabinogalactan Proteins Are the Possible Extracellular Molecules for Binding Exogenous Cerium(III) in the Acidic Environment Outside Plant Cells

**DOI:** 10.3389/fpls.2019.00153

**Published:** 2019-02-20

**Authors:** Qing Yang, Lihong Wang, Jingfang He, Haiyan Wei, Zhenbiao Yang, Xiaohua Huang

**Affiliations:** ^1^National and Local Joint Engineering Research Center of Biomedical Functional Materials, Jiangsu Collaborative Innovation Center of Biomedical Functional Materials, School of Chemistry and Materials Science, Nanjing Normal University, Nanjing, China; ^2^State Key Laboratory of Food Science and Technology, Jiangnan University, Wuxi, China; ^3^Center for Plant Cell Biology, Institute for Integrative Genome Biology, University of California, Riverside, Riverside, CA, United States

**Keywords:** cerium, arabinogalactan proteins, complex, structural basis, bind constant

## Abstract

Rare earth elements [REE(III)] increasingly accumulate in the atmosphere and can be absorbed by plant leaves. Our previous study showed that after treatment of REE(III) on plant, REE(III) is first bound by some extracellular molecules of plant cells, and then the endocytosis of leaf cells will be initiated, which terminates the endocytic inertia of leaf cells. Identifying the extracellular molecules for binding REE(III) is the crucial first step to elucidate the mechanism of REE(III) initiating the endocytosis in leaf cells. Unfortunately, the molecules are unknown. Here, cerium(III) [Ce(III)] and *Arabidopsis* served as a representative of REE(III) and plants, respectively. By using interdisciplinary methods such as confocal laser scanning microscopy, immune-Au and fluorescent labeling, transmission electron microscope (TEM), X-ray photoelectron spectroscopy (XPS), ultraviolet-visible spectroscopy, circular dichroism spectroscopy, fluorescent spectrometry and molecular dynamics simulation, we obtained two important discoveries: first, the arabinogalactan proteins (AGP) inside leaf cells were sensitively increased in protein expression and recruited onto the plasma membrane; second, to verify whether AGP can bind to Ce(III) in the acidic environment outside leaf cells, by choosing fasciclin-like AGP11 (AtFLA11) as a representative of AGP, we found that Ce(III) can form stable [Ce(H_2_O)_7_](III)-AtFLA11 complexes with an apparent binding constant of 1.44 × 10^−6^ in simulated acidic environment outside leaf cells, in which the secondary and tertiary structure of AtFLA11 was changed. The structural change in AtFLA11 and the interaction between AtFLA11 and Ce(III) were enhanced with increasing the concentration of Ce(III). Therefore, AtFLA11 can serve as Lewis bases to coordinately bind to Ce(III), which broke traditional chemical principle. The results confirmed that AGP can be the possible extracellular molecules for binding to exogenous Ce(III) outside leaf cells, and provided references for elucidating the mechanism of REE(III) initiating the endocytosis in leaf cells.

## Introduction

Rare earth elements [REE(III)] are comprised of the lanthanide elements (atomic number 57 to 71) together with scandium (atomic number is 21) and yttrium (atomic number is 39) in the periodic table of elements (Redling, 2006). With the ever-increasing update of the knowledge about the specific properties of REE(III) in physics, chemistry and biology, the applications of REE(III) are unprecedentedly increased in the field of energy (Bae et al., 2017; Tou et al., 2017), materials (Adesina et al., 2017; Hammond et al., 2017; Zhong et al., 2017), catalysis (Kim et al., 2016; Gollwitzer et al., 2017), biology (Aciego et al., 2017), environment (Aciego et al., 2017), etc., As a result of these applications, REE(III) have been released and widely accumulated in global environment in a large quantity (Shaltout et al., 2013; Hao et al., 2016; Paye et al., 2016). REE(III) accumulated in the environment inevitably makes contact with plants in the ecosystem. REE(III) as a series of non-essential heavy metal elements for living organisms affect the growth of plants (Gonzalez et al., 2014). For example, it was reported that REE(III) can improve the germination, photosynthesis, biomass, uptake of nutrition, dry weight of plants (Ramírezolvera et al., 2018; Vilela et al., 2018), and also exhibit toxic effects on plants (Joonas et al., 2016; Rezaee et al., 2018). More seriously, plants are the primary producers of the ecosystem, and can absorb and accumulate REE(III) from water, soil and atmosphere (Wang et al., 2014a; Zhuang et al., 2017). Consequently, REE(III) could be further transferred into human bodies through the food chain. It was recently reported that REE(III) in human body would cause serious injuries, such as the breakdown of the blood-brain barrier (Sweeney et al., 2018; Tur et al., 2018). Therefore, for protecting human health and the security of the ecosystem, it is urgent to establish the standard of the limit concentration of REE(III) in the ecosystem. To achieve this goal, it is necessary to clarify the mechanism of the biological effects of REE(III) on plants.

The biological effects of REE(III) on plants were discovered in 1917 (Chien and Ostenhout, 1917) and the mechanism of the biological effects of REE(III) on plants have been investigated at different levels in the past century (Huang et al., 2013; Wang et al., 2014b,c, 2017a; Yang et al., 2015; Zhang et al., 2018). However, the cellular and molecular mechanisms are still unclear. In 2014, by using interdisciplinary techniques, our group first reported a unique phenomenon after REE(III) in the atmosphere made contact with plant leaves: REEs(III) are first bound outside plant leaf cells, and then induce the initiation of the endocytosis in leaf cells, in which the endocytosis inertia of leaf cells resulted from long-term evolution is terminated. And the initiation of the endocytosis is the foremost unique response of plant cells to REE(III) (Wang et al., 2014a). This is the foremost response of plant cells to REE(III) that cannot be triggered by other pollution factors. Therefore, we think that this unique response is the key to investigate the unique biological effects of REE(III) on plants. To date, there is a serious lack of the knowledge about the endocytosis in plant cells due to the short of relevant investigations (Gadeyne et al., 2014; Fan et al., 2015; Zhang and Gao, 2015). In the investigations on the endocytosis in animal cells, endocytosis is usually classified into phagocytosis (≥250 nm) and pinocytosis (<250 nm) according to the diameter of endocytic vesicle (Pastan and Willingham, 1985). Our subsequent studies further discovered that the REE(III)-bound sites outside leaf cells and the type of the initiated endocytosis in leaf cells depend on the type of REE(III). For example, after different REE(III) make contact with plant leaves, cerium(III) [Ce(III)] is mainly bound on the plasma membrane (Yang et al., 2016b), and then initiates the pinocytosis in leaf cells (Wang et al., 2017c); lanthanum(III) and terbium(III) are bound in the extracellular matrix (Yang et al., 2016b), and then initiate the phagocytosis in plant cells (Wang et al., 2014a). More importantly, this unique endocytosis in plant leaf cells opens an unprecedented door for a large amount of REE(III) and other hazard substances directly entering and accumulating in plant cells (Wang et al., 2014a), and further entering human bodies through the food chain. Therefore, the initiated endocytosis in plant leaf cells responding to REE(III) in the atmosphere is not only harmful to plants, but also threatens human beings and the ecosystem. For these reasons, our work attracts increasing attention from interdisciplinary researchers who are appealing for establishing the standard for the limit concentration of REE(III) in the ecosystem (Vorob’ev et al., 2016; Garcíajiménez et al., 2017; Jin et al., 2017; Li et al., 2017).

To achieve this goal, the primary work is clarifying how REE(III) initiates the endocytosis of leaf cells. REE(III) as non-essential elements for plants must interact with some biological molecules in plants to make plants initiate the endocytosis in leaf cells. The biological molecules binding to REE(III) outside plant cells are the molecules primarily interacting with REE(III), which may be pivotal in the initiation of the endocytosis in leaf cells responding to REE(III). Therefore, determining the extracellular molecules for binding to exogenous REE(III) outside plant cells is the key for elucidating the mechanism of REE(III) initiating of the endocytosis in leaf cells.

Cerium(III) is the most abundant REE(III) in earth crust (Redling, 2006) and is widely used in the field of material, catalysis, medicine, etc., (Perez et al., 2016; Jiang et al., 2017; Liang et al., 2017). Consequently, Ce(III) accumulates in the global atmosphere as a contaminant of emerging concern with the content up to 917.30 mg kg^−1^ in PM2.5 (Yonemochi et al., 2016; Wang et al., 2017d; Zhao et al., 2017). Meanwhile, our previous studies indicated that the binding sites of Ce(III) outside plant leaf cells are much less than those of other REE(III) [such as lanthanum(III) and terbium(III)] (Wang et al., 2014a; Yang et al., 2016b; Wang et al., 2017c). Thus the molecules for binding Ce(III) outside leaf cells are more suitable for binding other REE(III). In this study, Ce(III) and *Arabidopsis* was chosen as a typical representative of REE(III) and plants, respectively. We used interdisciplinary methods involved in biophysics [confocal laser scanning microscopy (CLSM), transmission electron microscope (TEM) and X-ray photoelectron spectroscopy (XPS)], immunology (immune-Au and immune fluorescent labeling), spectroscopy [ultraviolet-visible spectroscopy (UV-vis), circular dichroism spectroscopy (CD) and fluorescent spectrometry (FL)] and computer science (molecular dynamics simulation) in the purpose of finding possible extracellular molecules of leaf cells for binding exogenous REE(III). The results will provide references for investigating the molecular and structural mechanisms of the initiation of the endocytosis in plant leaf cells responding to REE(III) for establishing the standard for the limit concentration of REE(III) in the ecosystem.

## Materials and Methods

### Chemicals and Experimental Materials

Ce_2_O_3_ (with the purity of 99.99%) was purchased from Sigma-Aldrich Company Ltd., (United States). We prepared CeCl_3_ solutions using the method we reported previously (Yang et al., 2016a): Ce_2_O_3_ was first dissolved in hydrochloric acid (HCl). After the excess HCl was evaporated, the Ce(III) mother solution was prepared after the dissolution of the residue in 1 M HCl and the subsequent dilution by distilled water. Then the concentration of Ce(III) in the mother solution was determined by complexometric titration (Cheng et al., 1965). Ethylene diamine tetraacetic acid and xylenol orange was utilized for titrating Ce(III) and serving as the indicator, respectively, and the concentration of Ce(III) in the mother solution was determined to be 200.00 mM. The solutions of Ce(III) for our experiments were prepared by diluting the mother solution of Ce(III) to the experimental concentrations (30 and 80 μM), and then the pH value of the solutions was adjusted to 6.0 by 0.1 M HCl. Ce(III) reportedly exists in the main form of [Ce(H_2_O)_8_](III) in the solution with a pH value less than or equal to 6.5 (Díazmoreno et al., 2011; Rudolph and Irmer, 2014).

**FIGURE 1:**
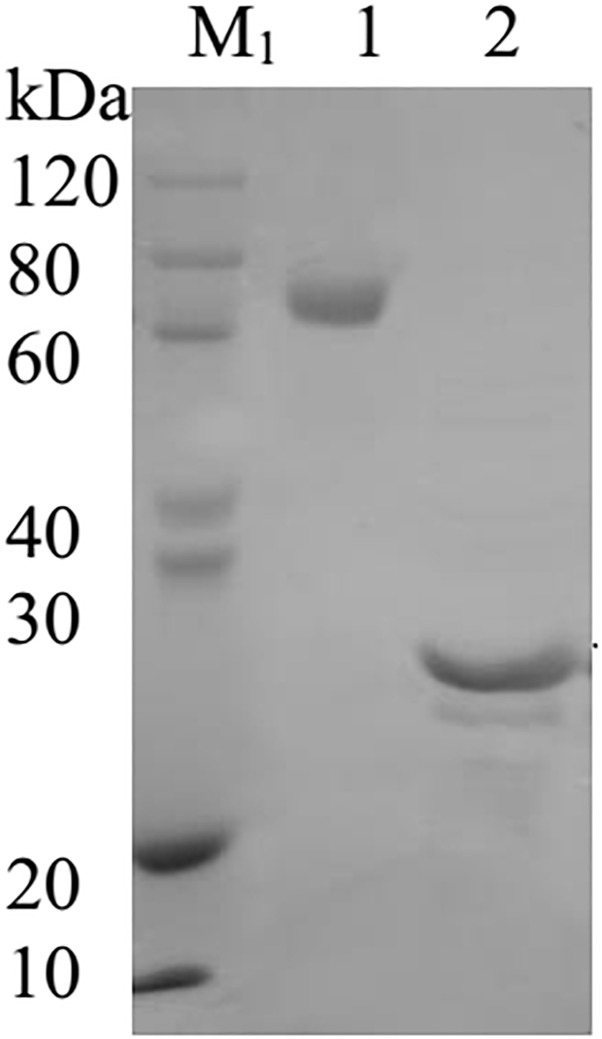
The image of the sodium dodecyl sulfate-polyacrylamide gel electrophoresis of AtFLA11. M_1_, SDS-PAGE Protein Marker; Lane 1, BSA protein; Lane 2, AtFLA11 protein.

The AtFLA11 [The *Arabidopsis* Information Resource (TAIR) AT5G03170.1] used in this study was synthesized and purified by Biomatic Co., (Canada) after expressing the protein in *E. Coli*. Therefore, the AtFLA11 was not expected to show the extensive post-translational modifications such as glycosylation, which may affect the protein functions *in vivo*. The AtFLA11 was preserved in the condition of −80°C. According to the result of sodium dodecyl sulfate-polyacrylamide gel electrophoresis provided by Biomatic Co., (Figure 1), the purity of AtFLA11 was > 90% (by calculating the gray value using Image J software, National Institutes of Health). The JIM13 antibody (rat source) was purchased from CarboSource Co., (United States). London Resin White (LR White) was purchased from Head Biotechnology Co., Ltd., (China). Rabbit Anti-Rat IgG (whole molecule)-gold (10 nm) antibody (Anti-IgG-Au) and *N*-(3-triethylammoniumpropyl)-4-[6-(4-diethylamino phenyl) hexatrienyl] pyridinium dibromide (FM4-64, 99% purity) were purchased from Sigma-Aldrich Company Ltd., (United States). Goat Anti-Rat IgG H&L (FITC) was purchased from Abcam Co., (United States). The purity of all other reagents was analytical grade.

### Plant Culture and Treatment

The *Arabidopsis thaliana* L. ecotype used in this study was Columbia 0 (Col-0). After sterilization by 1 mL 5% NaClO containing 0.1% Triton-X100 for 15 min, and wash by double-distilled water five times, the seeds of Col-0 were stored at 4°C for 48 h. The seeds were then sowed in Murashige and Skoog medium (containing 0.44% vitamin, 1% sucrose, and 0.8% agar) and were germinated at 22°C. The seedlings of Col-0 grew under the condition of 22°C, 60% humidity and 16 h:8 h (light:dark) photoperiod. The experimental solution of Ce(III) (30 or 80 μM) was sprayed onto the leaves of 20-days-old seedlings until the drop began to fall. Double-distilled H_2_O was sprayed onto the seedling leaves in the control group instead. 12 h later, we selected the leaves at a similar leaf position of each treatment group for further determination.

### Pinocytosis Observation

To observe the pinocytosis in leaf cells, the hypodermis from the selected leaves was pealed and incubated in FM4-64 solution (10 μM) for 30 min. Then, the pinocytosis in hypodermis cells was observed using a CLSM (Leica TCS SP8, Leica, 20×). The excitation and observation wavelength was 514 and 650 nm, respectively. There were five replicates for each treatment group, and we took 20 photos for each replicate in every treatment group. The average number of pinocytic vesicles in a leaf cell of each treatment group was calculated, and we chose the photos with the number of pinocytic vesicles at average levels for exhibiting in this paper.

### Immune-Au Labeling of Arabinogalactan Proteins (AGP)

We labeled the AGP in the leaf cells by immune-Au according to the method reported previously (Gao et al., 1999). After the sterilization with 70% ethanol for 2–3 min and wash with distilled water, the selected leaves were cut into strips with length of 2–3 mm. The leaf strips were fixed via the infiltration into a fixative solution (50 mM sodium cacodylate buffer containing 2% paraformaldehyde and 1% glutaraldehyde, pH 6.8) for 2 h at 4°C. After wash for 10 min by a 50 mM sodium cacodylate buffer ( × 5), the samples were dehydrated via being infiltrated into a series of ethanol solutions [30, 50, 70, 85, and 95% ethanol for 10 min, then 100% ethanol ( × 3) for 10 min]. To obtain the sample blocks, we successively infiltrated the dehydrated samples into ethanol/LR White mixture (3:1, 1:1 and 1:3, ethanol:LR White) for 30 min and 100% LR White ( × 2) for 60 min, followed by 100% LR White ( × 2) for 2 days. Finally, the samples were loaded in capsules which then were filled with LR White. The capsules were heated at 60°C for 24 h for polymerization. By using a Reichert-Jung Ultracut E Ultramicrotome (United States), the polymerized sample blocks were cut into ultrathin sections with the thickness of 60–70 nm, and then the ultrathin section put onto a nickel mesh, respectively.

To immune-label the AGP in the samples, the loaded nickel meshes were first washed using T1 buffer (50 mM sodium cacodylate buffer, pH 6.8, containing 2.5% NaCl, 0.1% bovine serum albumin, 0.05% Tween 20) ( × 5) for 5 min. After the incubation of the samples in the JIM13 solution (JIM13 was dissolved in T1 buffer, 1:10, v:v) at 4°C for 12 h, the samples was washed using T1 buffer ( × 3) for 10 min and T2 buffer ( × 2, 50 mM sodium cacodylate buffer, pH 6.8, containing 2% NaCl, 0.1% BSA, and 0.05% Tween 20) for 10 min. Then the samples were incubated in an Anti-IgG-Au solution (Anti-IgG-Au was dissolved in T2 buffer) at room temperature for 2 h. Finally, the samples were washed with T2 buffer ( × 5) for 5 min and dried. The samples were observed by using a TEM (HITACH H-7650). There were five replicates for each treatment group, and we took 20 photos for each replicate in every treatment group. The average quantity of nano-Au particles in a leaf cell of each treatment group was calculated, and we chose the photos with the quantity of nano-Au particles at average levels for exhibiting in this paper.

### Immune Fluorescent Labeling of AGP

To stain AGP using immune fluorescent dye, the hypodermis from the selected leaves was pealed and incubated in the solution of JIM13 (the antibody of AGP, rat source, 1:50) for 2 h. Then the samples were incubated in the solution of Goat Anti-Rat IgG H&L (FITC) (1:100) for 2 h. Then the samples were observed using a CLSM (Leica TCS SP8, Leica, 20 × ). The excitation and observation wavelength was 493 and 528 nm, respectively. There were five replicates for each treatment group, and we took 20 photos for each replicate in every treatment group. We chose the photos with the fluorescent signal at average levels for exhibiting in this paper.

### Interaction Between Ce(III) and AtFLA11 *in vitro*

AtFLA11 was first unfrozen at 25°C and then dissolved in the sodium cacodylate buffer to the concentration of 4.00 × 10^−6^ M. Next, Ce(III) in specific amount was added into AtFLA11 solutions to the molar ratios of [AtFLA11]:[Ce(III)] was 1:1, 1:2, 1:3, 1:4, 1:5, 1:6, 1:7, 1:8, 1:9, and 1:10, respectively. Then the AtFLA11 solution was diluted using sodium cacodylate buffer to the experimental concentrations. Finally, the solution pH value was adjusted to 6.0 using 1 × 10^−6^ M HCl. After incubating for 12 h at 4°C, the samples were prepared for further measurements.

### CD Measurement of AtFLA11

The CD measurements of AtFLA11 incubated with Ce(III) at different concentrations were performed using a Chirascan spectrometer (Applied Photophysics, United Kingdom) with differential spectrometry. Then the secondary structure of AtFLA11 was analyzed using CDNN software. The scanning speed was 100 nm min^−1^ and the scanning range was 180–280 nm. The results were exhibited in the form of molar ellipticity θ (deg cm^2^ d mol^−1^). The curves were plotted according to the average values of three replicates.

### FL Measurement of AtFLA11

The FL measurements of AtFLA11 incubated with Ce(III) at different concentrations were performed using an Ls-50B fluorescence photometer (Perkin-Elmer, Germany) with differential spectrometry. The excitation wavelength was 230 nm, the scanning range was 250–500 nm, and the scanning speed was 500 nm min^−1^. The curves were plotted according to the average values of three replicates.

### XPS Measurement of AtFLA11 and Ce(III)

The XPS measurements of AtFLA11 incubated with Ce(III) at different concentrations were performed by using an ESCALab MK2 X-ray photoelectron spectrophotometer (VG, East Grinstead, SXW, United Kingdom). The light source was a 225 W Mg Kα radiation. The XPS measurement was performed under 80 eV. The peak of C_1s_ (284.60 eV) was used for calibrating the binding energy. For preparing the samples, the solution containing AtFLA11 and Ce(III) was dropped onto the slides (0.8 × 0.8 cm). After they dried, the samples were measured under vacuum environments. There were three replicates for each treatment group, and the average binding energies were calculated. We chose the results with the binding energies at average levels for exhibiting in this paper.

### UV-vis Measurement of AtFLA11

The UV-vis spectra of AtFLA11 incubated with Ce(III) at different concentrations were recorded using a Cary 50 UV-vis spectrophotometer (Varian Medical Systems, Palo Alto, CA, United States) with differential spectrometry. The scanning wavelength range was 190–500 nm and the scanning speed was 240 nm min^−1^. The curves were plotted according to the average values of three replicates.

### Molecular Dynamic Simulation of the Interaction Between AtFLA11 and Ce(III)

The chemical calculations were performed using The Discovery Studios 2.5 package (DS 2.5, Accelrys, BIOVIA). After the calculation of the surface charge distribution of AtFLA11 (Supplementary Figure S2A), [Ce(H_2_O)_7_](III) was linked to a negatively charged area on the surface of AtFLA11 3D model to build a [Ce(H_2_O)_7_](III)-AtFLA11 model. The solvent was set to be water. After the optimization of the steepest descent and conjugate gradient methods, the molecular dynamics simulation was performed using DS 2.5. A result with the bond length of Ce(III)-O in normal range was selected for further analysis using DS 2.5 and Diamond 3.2 (Crystal Impact Inc., Bonn, Germany).

## Results

### Initiation of the Pinocytosis in Leaf Cells

The foremost unique response of plant leaf cells to Ce(III) bound outside leaf cells is the initiation of the pinocytosis in leaf cells (Wang et al., 2017c). FM4-64 is a fluorochrome that can serve as a marker of pinocytosis due to that FM4-64 can enter cells only via endocytosis (Bar et al., 2008; Kitakura et al., 2011; Lin et al., 2012). Therefore, FM4-64 is widely used in many studies to stain various cells (such as yeast and BY-2 cells) for different research aims (Lam et al., 2007; Balderhaar et al., 2013; Zheng et al., 2015). If the investigated cells (such as yeast and BY-2 cells) can initiate endocytosis in normal state, FM4-64 can enter these cells through endocytic routes (reaches early endosome and late endosome) (Lam et al., 2007; Balderhaar et al., 2013; Zheng et al., 2015). Consequently, the fluorescent signal of FM4-64 can be observed in these cells even in control groups (Lam et al., 2007; Balderhaar et al., 2013; Zheng et al., 2015). However, plant leaf cells have endocytic inertia in normal state, which is a result of long-term evolution (Buchanan et al., 2000). As a result, FM4-64 cannot enter normal leaf cells and we cannot observe the fluorescent signal of FM4-64 in normal leaf cells. If the pinocytosis is initiated in leaf cells after treatment of Ce(III), FM4-64 can enter leaf cells through pinocytosis (Wang et al., 2017c). Therefore, if we can observe the fluorescent signal of FM4-64 in the leaf cells treated with Ce(III), we can conclude that the pinocytosis in leaf cells is initiated after treatment of Ce(III).

**FIGURE 2:**
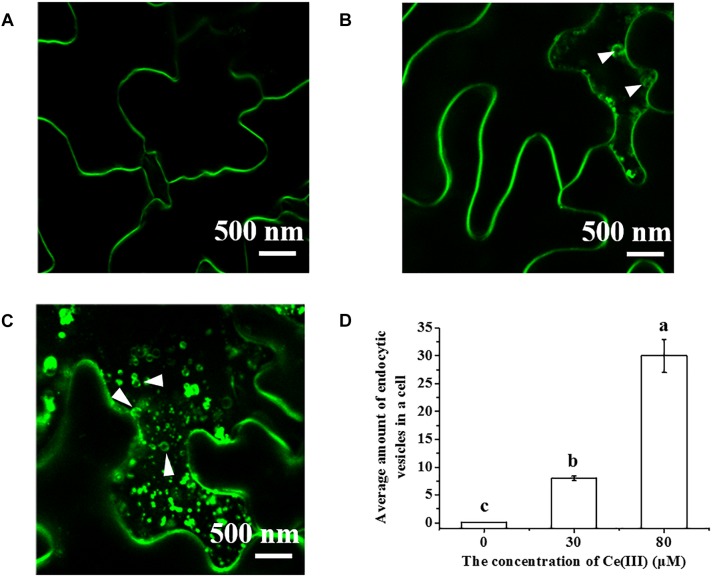
The CLSM images of FM4-64 labeled *Arabidopsis* hypodermis cells in the presence of Ce(III) at 0 **(A)**, 30 **(B)**, 80 μM **(C)** and the average amount of pinocytic vesicles in a leaf cell after Ce(III) treatments the different letter on the column means the values of the column are not significantly different at *p* < 0.05 **(D)**. The observation was carried out 12 h after the treatments. The pinocytic vesicles were pointed out by white arrows.

To identify the molecule binding to Ce(III) outside plant leaf cells, we first observed the pinocytosis in leaf cells labeled with FM4-64 in the presence of Ce(III) (Wang et al., 2017c). The results (Figure 2) showed that the boundary of the leaf cells treated without Ce(III) was smooth and clear, and there was no fluorescent vesicle in leaf cells (Figure 2A), indicating the pinocytosis inertia of leaf cells. This result is in accordance with the control group in the EMARG results we reported previously (Wang et al., 2017c). However, after treatment of 30 μM Ce(III), a few fluorescent vesicles with the diameters less than 250 nm emerged near the inside of the plasma membrane (pointed by white arrows in Figure 2B), indicating the initiation of the pinocytosis in leaf cells. After treatment of 80 μM Ce(III), more fluorescent vesicles near the inside of the plasma membrane emerged (pointed by white arrows in Figure 2C), indicating that the pinocytosis initiated in the leaf cells was enhanced. These results were in agreement with our previous EMARG results which showed that the pinocytosis in leaf cells can be initiated after treatment of ^141^Ce(III) and enhanced with increasing the concentration of ^141^Ce(III) (Wang et al., 2014a).

### Expression and Location of AGP in the Leaf Cells

**FIGURE 3:**
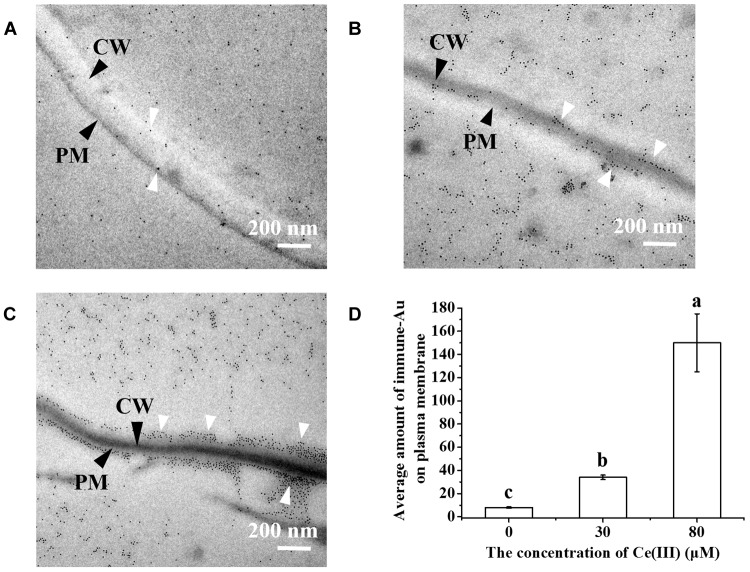
The TEM images of the immune-Au labeled AGP in *Arabidopsis* leaf cells in the presence of Ce(III) at 0 **(A)**, 30 **(B)**, 80 μM **(C)** and the average amount of immune-Au particles in a leaf cell after Ce(III) treatments the different letter on the column means the values of the column are not significantly different at *p* < 0.05 **(D)**. The observation was carried out 12 h after the treatments. Immune-Au labeled AGP were pointed by white arrows. CW, cell wall; PM, plasma membrane.

Arabinogalactan proteins are a series of highly glycosylated proteins, which are widely distributed outside plant cells as one of the main substances outside plant cells (Buchanan et al., 2000). AGP can regulate the cell growth, hormone response, signal transmission, etc., in the plant kingdom (Buchanan et al., 2000; Ellis et al., 2010; Nguema-Ona et al., 2013), and according to the investigations on animal cells, these cellular processes are closely related to endocytosis (Pierce et al., 2000; Gent et al., 2002; Colombani and Neufeld, 2006). We consequently determined the changes in the expression and subcellular location of AGP in the leaf cells treated with Ce(III) at different concentrations by immune-Au labeling AGP. The results (Figure 3) surprisingly showed that without the treatment of Ce(III), a few Au-nanoparticles distributed outside the plasma membrane (pointed by white arrows in Figure 3A), indicating the locations of a few AGP outside the plasma membrane, which is corresponding to the previous report (Gao et al., 1999). However, the treatment of 30 μM Ce(III) obviously increased the quantities of the AGP distributed outside and inside the plasma membrane of leaf cells (pointed by white arrows in Figure 3B); the treatment of 80 μM Ce(III) further significantly increased the quantities of AGP distributed outside and inside the plasma membrane of leaf cells (pointed by white arrows in Figure 3C).

**FIGURE 4:**
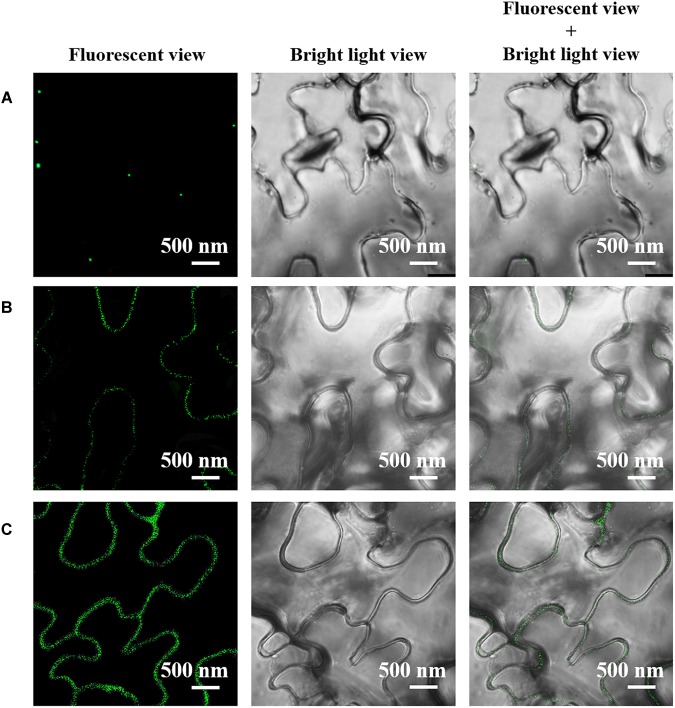
The CLSM images of the immune fluorescent labeled AGP in *Arabidopsis* hypodermis cells in the presence of Ce(III) at 0 **(A)**, 30 **(B)**, and 80 μM **(C)**. The immune fluorescent labeling was carried out 12 h after the treatments of Ce(III).

Meanwhile, we did the immune fluorescent labeling of AGP to further confirm the changes in the expression and subcellular location of AGP in the leaf cells treated with Ce(III) at different concentrations. The results (Figure 4) showed that without the treatment of Ce(III) a few AGP were located on the plasma membrane of leaf cells (Figure 4A). After the treatment of 30 μM Ce(III), the quantity of AGP on the plasma membrane was obviously increased (Figure 4B). After the treatment of 80 μM Ce(III), the quantity of AGP on the plasma membrane was further increased (Figure 4C). These results are in accordance with the results of immune-Au labeling (Figure 3).

All of these results indicated that with the initiation of the pinocytosis in leaf cells after treatment of Ce(III), the expression of AGP in leaf cells was promoted accompanied by the recruitment of AGP to outside leaf cells. As we know, once the structure and equilibrium concentration of AGP outside plant cells are disturbed or even destroyed, the AGP should be synthesized and supplemented from the inside to outside plant cells. Therefore, we speculated that AGP may bind to Ce(III) outside plant leaf cells to change the structure and equilibrium concentration of AGP. It therefore resulted in the promotion of AGP expression and the recruitment of AGP onto the plasma membrane of leaf cells to supplement AGP.

### Differential CD Spectra of AtFLA11

However, according to traditional knowledge, AGP can hardly bind to Ce(III) outside plant cells. According to chemical principle, REE(III) as Lewis acids can bind to Lewis bases to form stable Lewis acid-base complexes in alkaline or neutral environment (Huang, 2010). However, the environment outside plant cells is acidic (Buchanan et al., 2000), in which REE(III) generally cannot form stable Lewis acid-base complexes with Lewis bases (Huang, 2010). Therefore, we want to determine whether AGP can bind to Ce(III) in the acidic environment outside plant leaf cells.

Because of the complex environment and low content of biological molecules in the living system, it is very difficult to purify biological molecules. Therefore, there is a great difficulty in investigating the interaction between AGP and Ce(III) *in vivo*. AtFLA11 is a kind of AGP that has two AGP domains and one fasciclin-like domain (Cagnola et al., 2018) in *Arabidopsis*. AtFLA11 is mainly distributed outside plant cells, and plays important roles in regulating cell wall matrix integrity to contribute biomechanical properties (Macmillan et al., 2010; Cagnola et al., 2018). Meanwhile, the genetic expression of AtFLA11 also exhibited a little response to the treatment of Ce(III) (Supplementary Figure S3). Thus we chose AtFLA11 as a representative of AGP and simulated the interaction between AtFLA11 and Ce(III) *in vitro* in an acidic environment which is similar to the environment outside plant cells.

**FIGURE 5:**
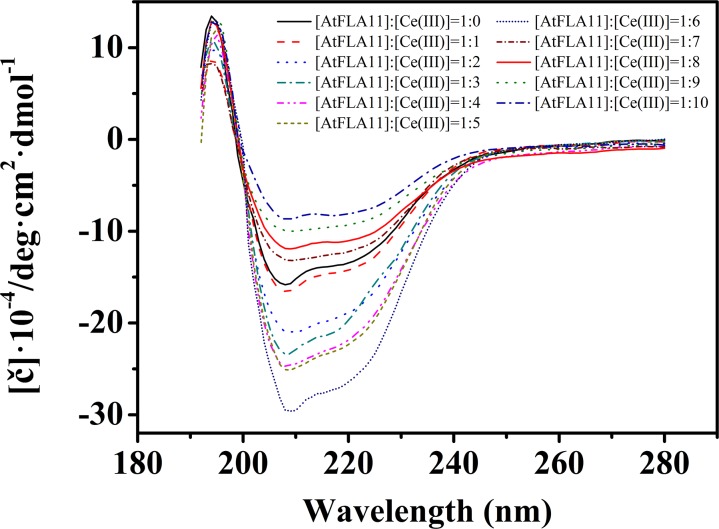
The circular dichroism spectra of 1.00 × 10^−6^ M AtFLA11 incubated with Ce(III) at different concentrations. The measurements were carried out 12 h after incubating AtFLA11 with Ce(III).

When a Lewis acid approaches a Lewis base via electrostatic attraction, they change the electron cloud distribution of each other. The change in the electron cloud distribution of a protein (Lewis base) will reform the H-bonds in the protein, and finally change the secondary structure of the protein. Therefore, to confirm the interaction between AtFLA11 and Ce(III), we first determined the change in the secondary structure of AtFLA11 by measuring the CD spectra of AtFLA11 incubated with Ce(III) at different concentrations. The results are exhibited in Figure 5. Without the incubation with Ce(III), a positive peak emerged near 194 nm in the CD spectra of AtFLA11, which was caused by the π-π^∗^ transition of the amide bands in AtFLA11. Meanwhile, the π-π^∗^ and n-π^∗^ transitions of the amide bands in AtFLA11 caused two negative peaks near 209 and 222 nm, respectively, which represented the α-helixes and β-sheets in AtFLA11 (Zhao, 2000). Compared to those of the control, when the [AtFLA11]:[Ce(III)] ratio ranged from 1:1 to 1:6, the absorbance near 194 nm was gradually increased, indicating a strengthening interaction between the amide groups in AtFLA11 and Ce(III). Meanwhile the absorbance at 209 and 222 nm was intensified with increasing the concentration of Ce(III), indicating the increase in the content of α-helixes or β-sheets in AtFLA11 (Zhao, 2000). When the [AtFLA11]:[Ce(III)] ratio ranged from 1:7 to 1:10, the absorbance near 194 nm was almost constant, indicating an constant interaction between the amide groups in AtFLA11 and Ce(III). It implied a dynamic equilibrium of the interaction between AtFLA11 and Ce(III). Meanwhile, the absorbance near 209 and 222 nm was weakened with increasing the concentration of Ce(III), indicating the decrease in the content of α-helixes and β-sheets in AtFLA11.

The studies of structural biology showed that a protein’s secondary structure consists of by ordered conformations [constructed by intra-chain H-bonds (α-helix) or inter-chain H-bonds (β-sheet and β-turn)] and disordered conformation (random coil) (Liljas et al., 2009). The secondary structures of AtFLA11 incubated with Ce(III) at different concentrations are listed list in Table 1. In comparison with the control, when the [AtFLA11]:[Ce(III)] ratio ranged from 1:1 to 1:6, the content of the α-helix in AtFLA11 was gradually increased by 9.4–15.0%, the content of the β-sheet in AtFLA11 was gradually decreased by 6.2–15.5%, while the content of the β-turn in AtFLA11 was not obviously changed. It indicated the formation of the intra-chain H-bonds and the disruption of the inter-chain H-bonds in AtFLA11 (Liljas et al., 2009). Meanwhile, the content of the random coil in AtFLA11 was decreased by 11.8–20.4% with increasing the concentration of Ce(III), indicating the decrease in the content of ordered the conformation in AtFLA11. When the [AtFLA11]:[Ce(III)] ratio ranged from 7 to 10, the content of the α-helix in AtFLA11 was gradually decreased by 19.9–25.4%, the content of the β-sheet in AtFLA11 was gradually increased by 19.9–27.4%, while the content of the β-turn in AtFLA11 was still basically unchanged. It indicated the disruption of the intra-chain H-bonds and the formation of the inter-chain H-bonds in AtFLA11 (Liljas et al., 2009). Meanwhile, the content of the random coil in AtFLA11 was increased by 21.3–29.3% with increasing the concentration of Ce(III), indicating the increase in the content of the disordered the conformation in AtFLA11. All of these results indicated that AtFLA11 can interact with Ce(III) in acidic environment, which resulted in the change in the secondary structure of AtFLA11.

**Table 1:** The secondary structure information of AtFLA11 incubated with different concentrations of Ce(III).

Condition	Content of α-helix (%)	Content of β-sheet (%)	Content of β-turn (%)	Content of random coil (%)
[AtFLA11]:[Ce(III)] = 1:0	28.70%	22.60%	18.00%	31.40%
[AtFLA11]:[Ce(III)] = 1:1	31.40%	21.20%	18.10%	27.70%
[AtFLA11]:[Ce(III)] = 1:2	31.80%	20.20%	17.90%	26.90%
[AtFLA11]:[Ce(III)] = 1:3	31.80%	19.90%	17.40%	26.20%
[AtFLA11]:[Ce(III)] = 1:4	31.90%	19.60%	17.40%	25.70%
[AtFLA11]:[Ce(III)] = 1:5	32.60%	19.50%	18.00%	25.30%
[AtFLA11]:[Ce(III)] = 1:6	33.00%	19.10%	18.30%	25.00%
[AtFLA11]:[Ce(III)] = 1:7	23.00%	27.10%	18.00%	38.10%
[AtFLA11]:[Ce(III)] = 1:8	22.50%	27.50%	18.20%	39.10%
[AtFLA11]:[Ce(III)] = 1:9	21.60%	28.00%	18.70%	39.80%
[AtFLA11]:[Ce(III)] = 1:10	21.40%	28.80%	17.60%	40.60%

### Differential FL Spectra of AtFLA11

**FIGURE 6:**
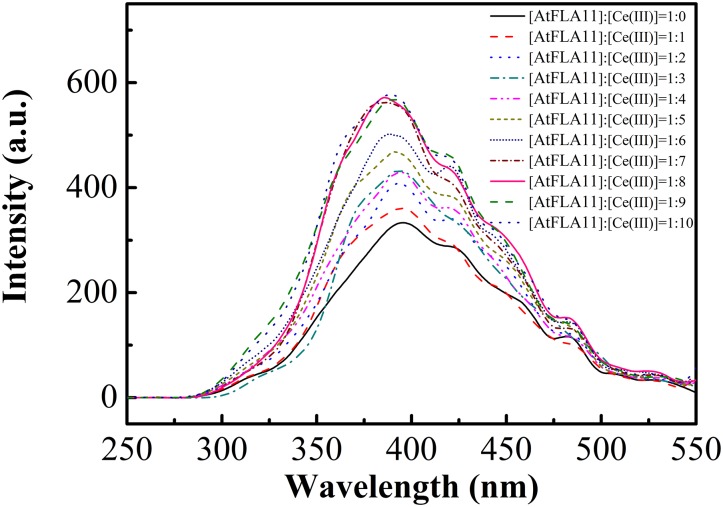
The fluorescence spectra of 1.00 × 10^−6^ M AtFLA11 incubated with Ce(III) at different concentrations. The measurements were carried out 12 h after incubating AtFLA11 with Ce(III).

In protein molecules, secondary structures are further folded into tertiary structures by a hydrophobic force. Would the changes in the secondary structure of AtFLA11 after interacting with Ce(III) further affect the tertiary structure of AtFLA11? To further comprehend the changes in the molecular structure of AtFLA11 after the interaction between AtFLA11 and Ce(III) in the acidic environment, the FL spectra of AtFLA11 incubated with or without Ce(III) was measured. The results are exhibited in Figure 6. In the FL spectra of AtFLA11 incubated without Ce(III), two absorption peaks emerged at 394 and 421 nm, respectively. These two peaks were caused by two hydrophobic amino acid residues—tyrosine and tryptophan, respectively (De and Girigoswami, 2006). When the [AtFLA11]:[Ce(III)] ratio ranged from 1:1 to 1:6, these two peaks did not shift. However, the intensities of these two peaks were gradually increased with increasing the concentration of Ce(III). These results indicated the increase in the exposures of tyrosine and tryptophan in AtFLA11. It implied the disruption of the hydrophobic force in AtFLA11, which represented the changes in the tertiary structure of AtFLA11. When the [AtFLA11]:[Ce(III)] ratio ranged from 1:7 to 1:10, the intensities of the two peaks maintained constant. It indicated the tertiary structure of AtFLA11 was not further changed, which implied a dynamic equilibrium of the interaction between AtFLA11 and Ce(III), and it is in accordance with the results of CD spectra (Figure 5). These results indicated that after the interaction between AtFLA11 and Ce(III) in the acidic environment, the changes in the secondary structure of AtFLA11 further changed the tertiary structure of AtFLA11.

### XPS of AtFLA11 and Ce(III) Before and After the Incubation

**FIGURE 7:**
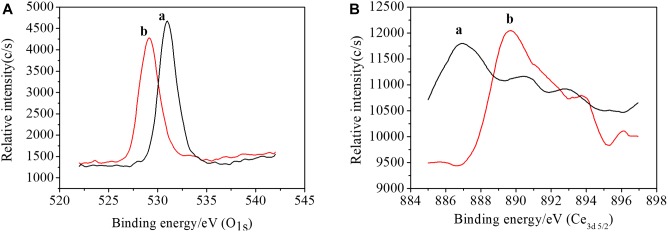
The XPS spectra of AtFLA11 with [(a), black line] or without [(b), red line] Ce(III). **(A)** O_1s_; **(B)** Ce_3d5/2_. The measurements were carried out 12 h after incubating AtFLA11 with Ce(III).

The interaction between AtFLA11 and Ce(III) would be intensified with Ce(III) approaching AtFLA11. Meanwhile, the results of CD and FL spectra indicated the gradually strengthened interaction between AtFLA11 and Ce(III) with increasing the concentration of Ce(III). Therefore, a further question is: Can AtFLA11 bind to Ce(III) to form Ce(III)-AtFLA11 complexes in the acidic environment? Answering this question is a great challenge to traditional chemical principle (Huang, 2010). To confirm whether AtFLA11 can bind to Ce(III) to form Ce(III)-AtFLA11 complexes in acidic environment, we recorded the XPS spectra of AtFLA11 and Ce(III) before and after the incubation of AtFLA11 with Ce(III). The results are exhibited in Figure 7 and Table 2. Before the incubation of AtFLA11 with Ce(III), the average binding energy of O_1s_ in AtFLA11 and Ce_3d5/2_ was 529.13 (Figure 7A and Table 2) and 893.70 eV (Figure 7B and Table 2), respectively, which approximated to the reported values (Wang et al., 2010). After the incubation of AtFLA11 with Ce(III), the average binding energy of O_1s_ in AtFLA11 was increased to 531.00 eV (Figure 7A and Table 2), while the average binding energy of Ce_3d5/2_ was decreased to 886.95 eV (Figure 7B and Table 2). It indicated the decrease in the electron cloud density of O and the increase in the electron cloud density of Ce(III) (Wang et al., 2010). Therefore, the O in AtFLA11 bound to Ce(III) to form Ce(III)-AtFLA11 complexes in acidic environment. Combined the results of CD spectra (Figure 5), we confirmed that the O in the amide groups in AtFLA11 served as the Lewis base and electron donor to bind to Ce(III), which served as Lewis acid and electron receptor. Thus the electron cloud in the O shifted to Ce(III), and finally increased the average binding energy of O_1s_ and decreased the average binding energy of Ce_3d5/2_. In addition, the decrease in the average orbital binding energy of Ce_3d5/2_ was much greater than those of some other REE(III) bound to proteins (Wang et al., 2016, 2017b), indicating a stronger binding between AtFLA11 and Ce(III). Therefore, AtFLA11 can serve as Lewis bases to bind to Ce(III) to form stable Ce(III)-AtFLA11 complexes in an acidic environment.

### Differential UV-vis Spectra of AtFLA11 in the Presence of Ce(III)

**FIGURE 8:**
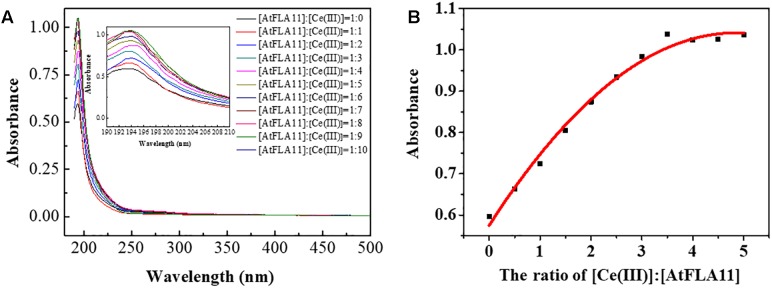
The UV-vis spectra of 5.00 × 10^−7^ M AtFLA11 containing Ce(III) at different concentrations **(A)** and the fitted curve of the absorbance near 194 nm **(B)**. The measurements were carried out 12 h after incubating AtFLA11 with Ce(III).

The above-mentioned results obtained from CD spectra, FL spectra and XPS indicated that the interaction between AtFLA11 and Ce(III) was gradually strengthened with increasing the concentration of Ce(III), and AtFLA11 finally bound to Ce(III) to form Ce(III)-AtFLA11 complexes in the acidic environment. To further verify the interaction between AtFLA11 and Ce(III) in the acidic environment, the UV-vis spectra of AtFLA11 incubated with Ce(III) at different concentrations were recorded. The results are exhibited in Figure 8A. Before the incubation with Ce(III), a peak emerged near 194 nm in the UV-vis spectra of AtFLA11. It represented the π-π^∗^ transition of the amide groups in AtFLA11 (Guo et al., 2008; Wang et al., 2016), which was in accordance with the results of CD spectra (Figure 5). After the incubation with Ce(III), the peak shift was not observed. When the [AtFLA11]:[Ce(III)] ratio ranged from 1:1 to 1:6, the absorbance near 194 nm was enhanced with increasing the concentration of Ce(III). Combined with the results of XPS (Figure 7 and Table 2), it indicated the strengthening interaction between the O in the amide groups in AtFLA11 and Ce(III), which was in agreement with the results of CD spectra (Figure 5). When the [AtFLA11]:[Ce(III)] ratio ranged from 1:7 to 1:10, the absorbance near 194 nm was not obviously changed, indicating the dynamic equilibrium of the interaction between Ce(III) and AtFLA11. These results were in accordance with the results of CD spectra (Figure 5), and further confirmed the binding between Ce(III) and AtFLA11 in the acidic environment.

**Table 2:** The average orbital binding energies of C_1s_, O_1s_ and Ce_3d5/2_ in AtFLA11 incubated with Ce(III) at different concentrations.

Condition	C_1s_/eV	O_1s_/eV	Ce_3d5/2_/eV
[AtFLA11]:[Ce(III)] = 1:0	284.60	529.13	–
CeCl_3_	284.60	–	893.70
[AtFLA11]:[Ce(III)] = 1:10	284.60	531.00	886.95

Figure 8B shows the fitted curve of the absorbance near 194 nm, which also indicated that when the [AtFLA11]:[Ce(III)] ratio ranged from 1:0 to 1:6, the absorbance near 194 nm was intensified with increasing the concentration of Ce(III) in some linear relationship. When the [AtFLA11]:[Ce(III)] ratio ranged from 1:7 to 1:10, the absorbance near 194 nm attained was constant. The apparent binding constant of the interaction between AtFLA11 and Ce(III) was 1.44 × 10^−6^, which was calculated according to this fitted curve. This apparent binding constant is greater than the previously reported binding constant of REE(III) binding to some other proteins, such as vitronectin-like protein and bovine serum albumin (Wang et al., 2017b; Liang et al., 2008), indicating that AtFLA11 is a more preferential molecule for binding to Ce(III).

### Molecular Dynamic Simulation of the Binding Between AtFLA11 and Ce(III)

**FIGURE 9:**
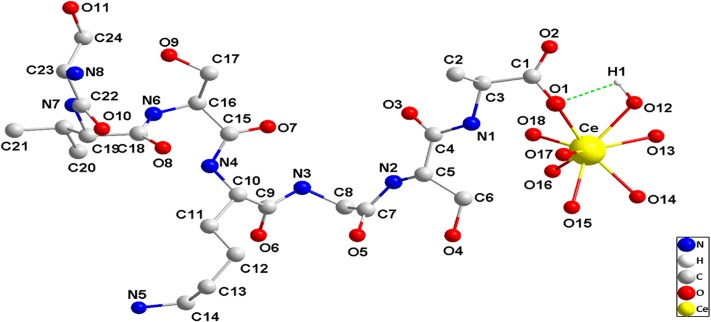
The partial schematic diagram of AtFLA11 coordinately bound to [Ce(H_2_O)_7_](III) to form [Ce(H_2_O)_7_](III)-AtFLA11 complex.

How does AtFLA11 bind to Ce(III) in the acidic environment outside leaf cells? Investigating the binding mode between AtFLA11 and Ce(III) can elucidate the mechanism of AtFLA11 binding to Ce(III) in the acidic environment at structural level. Because of the difficulty in purifying the Ce(III)-AtFLA11 complexes in plants and getting the crystal structure of the complexes (Smialowski and Frishman, 2010), it is hardly possible to investigate the binding mode between AtFLA11 and Ce(III) by experiments. Bioinformatics are flourishing in recent years, especially, biochemistry, mathematics and computational chemistry, which are rapidly developing (Blackwell, 2008). Thus modeling and a molecular dynamic stimulation operated by computers have become a reliable and effective method for predicting and stimulating the interactions between molecules (Nguyen and Hall, 2004; Tedesco et al., 2015). Therefore, we carried out molecular dynamic simulation to simulate the binding mode between AtFLA11 and Ce(III) in an acidic environment. Ce(III) was considered as in the form of [Ce(H_2_O)_7_](III) in the physiological environment (Yang et al., 2016a; Wang et al., 2017b). After building the 3D model of AtFLA11 (Supplementary Figures S1, S2), we performed a molecular dynamic simulation to simulate the binding mode between AtFLA11 and Ce(III), and the result is shown in Figure 9. The result exhibits that the carboxyl O (O1 in Figure 9) in the Ala (No. 206) in AtFLA11 can form a coordination bond with the Ce(III) in [Ce(H_2_O)_7_](III) to form a [Ce(H_2_O)_7_](III)-AtFLA11 complex. The bond length of Ce(III)-O was 2.2786 Å (Supplementary Table S1) which was shorter than the normal value (Xian and Lin, 2003). It indicated that AtFLA11 could coordinately bind to Ce(III) to form a stable [Ce(H_2_O)_7_](III)-AtFLA11 complex, which was in accordance with the results of XPS (Figure 7 and Table 2) and UV-vis spectra (Figure 8). Meanwhile, H in [Ce(H_2_O)_7_](III) can form a H-bond with a surrounding O in the length of 2.6709 Å (Supplementary Table S1) which belonged to a strong H-bond (Jin et al., 2013). It therefore increased the stability of the [Ce(H_2_O)_7_](III)-AtFLA11 complex. These results further verified that AtFLA11 can bind to Ce(III) in an acidic environment via forming a stable [Ce(H_2_O)_7_](III)-AtFLA11 complex, and provided a possible mode of AtFLA11 binding to Ce(III). Partial structural parameters of AtFLA11 before and after the binding to [Ce(H_2_O)_7_](III) are listed in Supplementary Table S1. The data indicated that all the parameters in AtFLA11 had been changed after the formation of the [Ce(H_2_O)_7_](III)-AtFLA11 complex, indicating the change in the molecular structure of AtFLA11. It was in accordance with the results of CD (Figure 5 and Table 1) and FL spectra (Figure 6).

## Discussion

The initiation of the pinocytosis in leaf cells as the unique response of leaf cells to the exogenous Ce(III) in the atmosphere is the key to explaining the special biological effects of REE(III) in the atmosphere on plants. Meanwhile, the initiation of the pinocytosis in leaf cells responding to exogenous Ce(III) in the atmosphere mainly depends on the molecules binding to Ce(III) outside plant leaf cells, which would play an important role in triggering the initiation of the pinocytosis in leaf cells. However, the traditional chemical principle indicates that Ce(III) as Lewis acid can hardly bind to Lewis bases to form stable Lewis acid-base complexes in the acidic environment outside plant leaf cells, and Lewis acid-base complexes are in the tendency of dissociation in the acidic environment (Huang, 2010). It gives a challenge for finding the possible extracellular molecules for binding Ce(III) outside leaf cells.

In this study, to our surprise, we discovered that when a plant initiated the pinocytosis in leaf cells responding to Ce(III) (Figure 2), the expression of AGP in leaf cells was simultaneously accelerated, accompanied by the recruitment of AGP onto the plasma membrane (Figure 3, 4). This response implied that AGP might bind to Ce(III) outside leaf cells. Then the structure and equilibrium concentration of AGP outside leaf cells would be disturbed or even destroyed. Consequently, leaf cells promoted the synthesis of AGP and supplemented the natural AGP to outside leaf cells. To confirm whether AGP are possible extracellular molecules for binding to Ce(III) outside leaf cell, it should verify that AGP can bind to Ce(III) in the acidic environment outside leaf cells, which is a great challenge to the traditional chemical principle (Huang, 2010). Therefore, we simulated the interaction between AGP and Ce(III) in an acidic environment similar to the environment outside plant cells.

The interaction between a Lewis acid and a Lewis base begins with their approaching each other via electrostatic attraction, during which the electron clouds of the Lewis acid and Lewis base will be increasingly changed (Miessler and Tarr, 2011). The H-bond is a type of typical weak force that widely exists in protein molecules and plays significant roles in maintaining the molecular structure of proteins (Liljas et al., 2009). According to the principle of similarity and intermiscibility, the peptide chain of protein is first folded to construct the secondary structure of protein via forming intra-chain H-bonds (α-helixes) and inter-chain H-bonds (β-sheet and β-turn) (Rehm, 2007). Based on this, the secondary structure of protein is further folded to construct the tertiary structure of protein via a hydrophobic force, which can wrap most of the hydrophobic groups into a protein molecule (Rehm, 2007). The change in the electron cloud can easily result in the reformation of the H-bonds in proteins, which then change the molecular structure of proteins.

The results of CD spectra verified that when Ce(III) (Lewis acid) and AtFLA11 (Lewis bases) approached each other in the acidic environment similar to the environment outside plant cells, the reformation of the H-bonds in AtFLA11 took place. It therefore disturbed the secondary structure of AtFLA11 (Figure 5 and Table 1). With increasing the concentration of Ce(III), the interaction between AtFLA11 and Ce(III) was gradually strengthened, and exhibited different effects on the secondary structure of AtFLA11 (Figure 5). The reformation of H-bonds in the presence of Ce(III) at low concentrations increased the intra-chain H-bonds and decreased the inter-chain H-bonds in AtFLA11. Therefore, it increased the content of α-helix and decreased the content of β-sheet (Table 1). Meanwhile, it also decreased the content of random coil, indicating the decrease in the disordered the conformation (Table 1). Consequently, Ce(III) at low concentration may make the structure of AtFLA11 more ordered. With increasing the concentration of Ce(III), the charge distribution of AtFLA11 was severely changed, which made massively disrupted the H-bond reformation in the intra-chain H-bonds in AtFLA11, and therefore significantly decreased the content of α-helix in AtFLA11 (Table 1). Although the reformation of a H-bond increased the content of β-sheet in AtFLA11, the content of the disordered conformation in AtFLA11 was increased by more than 5% (Table 1), which was greater than the extent that can be self-repaired (Liljas et al., 2009). It therefore indicated the irreversible change in the molecular structure of AtFLA11, which made the conformation of AtFLA11 was irreversibly loosened.

The results of FL spectra confirmed that the changes in the secondary structure of AtFLA11 in the presence of Ce(III) would further affect the intracellular hydrophobic force in AtFLA11. With increasing the concentration of Ce(III), the exposure of the hydrophobic groups in AtFLA11 was gradually enhanced (Figure 6). Combined with the results of CD spectra (Figure 5 and Table 1), although the exposure of the hydrophobic groups in AtFLA11 was increased by the treatments of Ce(III) at low concentrations, the content of the ordered conformation in AtFLA11 was increased. Therefore, the tertiary structure of AtFLA11 was just moderately modulated. With increasing the concentration of Ce(III), the secondary structure of AtFLA11 was disrupted. It consequently disrupted the intracellular hydrophobic force in AtFLA11, which made the peptide chain of AtFLA11 unable to be correctly folded, and therefore resulted in greater exposure of the hydrophobic groups (Figure 6).

To serve as the possible molecules for binding to Ce(III) outside leaf cells, AtFLA11 must strongly interact with Ce(III) in the acidic environment, that is, AtFLA11 must form stable Ce(III)-AtFLA11 complexes with Ce(III) in the environment. The results of FL spectra indicated that the hydrophobicity on the molecular surface of AtFLA11 was gradually increased with the enhancement of the interaction between AtFLA11 and Ce(III) (Figure 6). It therefore improved the environment for AtFLA11 binding to Ce(III) in the acidic environment. The results of XPS (Figure 7 and Table 1) and UV-vis spectra (Figure 8) jointly verified that the O in the amide group in AtFLA11 can serve as Lewis base to interact with Ce(III) in acidic environment, and the strength of the interaction was enhanced with increasing the concentration of Ce(III) (Figure 7, 8 and Table 1). Finally, AtFLA11 bound to Ce(III) to form Ce(III)-AtFLA11 complexes in the acidic environment. It consequently indicated that AtFLA11 can bind to Ce(III) in the acidic environment, which broke traditional chemical principle. The apparent binding constant of the interaction was 1.44 × 10^−6^ (Figure 8B), which was much greater than those of the interaction between REE(III) and some other proteins (Wang et al., 2017b; Liang et al., 2008).

The results of molecular dynamic stimulation elucidated a probable binding mode between AtFLA11 and Ce(III) at structural level. The results indicated that at least the O in the alanine (Ala) (No. 206) in AtFLA11 could bind to Ce(III) in [Ce(H_2_O)_7_](III) to form [Ce(H_2_O)_7_](III)-AtFLA11 complex in acidic environment (Figure 9), which further confirmed the results of XPS (Figure 7 and Table 2). The bond length of Ce-O was shorter than the normal range of Ce-O reported before (Supplementary Table S1) (Xian and Lin, 2003), indicating the strong interaction between AtFLA11 and Ce(III). This result validated the apparent change in the binding energy of Ce_3d5/2_ (Table 2) and the great binding constant calculated according to the UV-vis spectra (Figure 8B). Meanwhile, the H in [Ce(H_2_O)_7_](III) could form a strong H-bond with some other O in AtFLA11 (Figure 9 and Supplementary Table S1), and therefore enhanced the stability of the [Ce(H_2_O)_7_](III)-AtFLA11 complex in the acidic environment (Figure 9). All of the above-mentioned results indicated that AtFLA11 can bind to Ce(III) to form stable [Ce(H_2_O)_7_](III)-AtFLA11 complexes in the acidic environment. After the formation of the [Ce(H_2_O)_7_](III)-AtFLA11 complex, all the structural parameters in AtFLA11 were changed (Supplementary Table S1). It was indicative of the change in the secondary and tertiary structure of AtFLA11, which was in accordance with the results of CD (Figure 5) and FL spectra (Figure 6).

**FIGURE 10:**
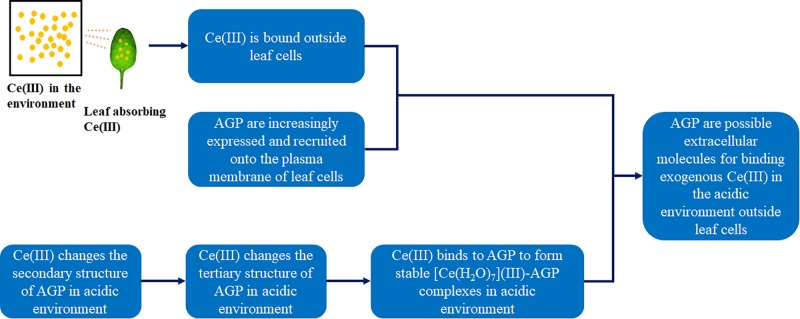
The confirmation of AGP being the possible extracellular molecules for binding to exogenous Ce(III) outside leaf cells.

Because most of AGP molecules have similar negatively charged groups, we think that other kinds of AGP can also bind to Ce(III) in the acidic environment. Therefore, AGP can be the possible extracellular molecules for binding to exogenous Ce(III) outside leaf cells. Figure 10 shows the confirmation of AGP being the possible extracellular molecules for binding to exogenous Ce(III) outside leaf cells. In addition, there are very complicated environments in the cells of living organisms for maintaining complex life processes. To date, it is still a great difficulty to elucidate the structural mechanisms of the biological reaction in organisms. Therefore, the results of this study will be verified by the experiment *in vivo* in the future.

## Conclusion

In this study, we found that with the initiation of the pinocytosis in leaf cells treated with Ce(III), AGP in leaf cells was increasingly expressed and recruited onto the plasma membrane. Meanwhile, after AGP interacts with Ce(III) in the acidic environment similar to the environment outside leaf cells, Ce(III) first changes the electron cloud distribution of AGP, which then reformed the H-bonds in AGP. Therefore, Ce(III) disturbed the secondary structure of AGP. Ce(III) at low concentration decreased the random coil content in AGP, which made the molecular structure of AGP more orderly; while Ce(III) at high concentration increased the random coil content in AGP, which made the molecular structure of AGP more loose. The changes in the secondary structure of AGP further disrupted the hydrophobic force in AGP and consequently increased the exposure of the hydrophobic groups, which therefore changed the tertiary structure of AGP. The structural change in AGP was enhanced with increasing the concentration of Ce(III). The increase in the hydrophobicity on the molecular surface of AGP improved the environment for AGP binding to Ce(III) in the acidic environment on the plasma membrane of plant leaf cells. The O of the amide groups in AGP served as Lewis bases to coordinately bind to Ce(III), which was Lewis acid, with an apparent binding constant of 1.44 × 10^−6^. Therefore, AGP formed stable [Ce(H_2_O)_7_](III)-AGP complexes with Ce(III) in the acidic environment. Meanwhile, the H-bond between the H in [Ce(H_2_O)_7_](III) and a surrounding O in AGP stabilized the [Ce(H_2_O)_7_](III)-AGP complexes. Therefore, AGP can be the possible molecules for binding to exogenous Ce(III) in the acidic environment outside leaf cells. Our results broke traditional chemical principle to update the current knowledge about the interaction between biological Lewis acid and base in acidic environment, and provided references for elucidating the mechanism of the initiation of the endocytosis in leaf cells responding to REE(III) for establishing the standard for the limit concentration of REE(III) in the ecosystem.

## Author Contributions

XH conceived the study and designed the experiments. JH, QY, and LW carried out the experiments. QY and HW did the molecular dynamics simulation. QY, LW, JH, HW, ZY, and XH analyzed the data. QY, LW, JH, and XH wrote the manuscript.

## Conflict of Interest Statement

The authors declare that the research was conducted in the absence of any commercial or financial relationships that could be construed as a potential conflict of interest.
