# Supplementary material for: Arabinogalactan Proteins Are the Possible Extracellular Molecules for Binding Exogenous Cerium(III) in the Acidic Environment Outside Plant Cells
Source: Front Plant Sci. 2019 Feb 20;10:153. doi: 10.3389/fpls.2019.00153 (PMC6391350; doi:10.3389/fpls.2019.00153)
Supplement: Supplementary file 1 [file Data_Sheet_1.pdf]

SUPPLEMENTARY

Arabinogalactan Proteins are the Possible Extracellular Molecules for Binding Exogenous Cerium(III) in the Acidic Environment outside Plant Cells

Qing Yang [1†], Lihong Wang [2†], Jingfang He [1], Haiyan Wei [1], Zhenbiao Yang [3], Xiaohua Huang [1, *]

[1] *National and Local Joint Engineering Research Center of Biomedical Functional Materials, Jiangsu Collaborative Innovation Center of Biomedical Functional Materials, School of Chemistry and Materials Science, Nanjing Normal University, Nanjing 210046, China*

[2] *State Key Laboratory of Food Science and Technology, Jiangnan University, Wuxi 214122, China*

[3] *Center for Plant Cell Biology, Institute for Integrative Genome Biology, University of California, Riverside, CA 92521, USA*

[†] These authors contributed equally to this paper.

* Corresponding author. Email:

huangxiaohuanjnu@yahoo.com (X. Huang)

Table S1 The structural indices (bond lengths, bond angles and torsion angles) of the part in ATFLA11 3D model (yellow part in Fig. S2B) and the complex of part-Ce(III) at pH 6.0.

	Part	Part-Ce
Bond length (Å)		
C1-C2	1.5605	1.5414
C2-C3	1.5633	1.6071
C4-C5	1.4898	1.5321
C5-C6	1.5913	1.5028
C7-C8	1.4923	1.5095
C9-C10	1.5111	1.5601
C10-C11	1.4998	1.5728
C11-C12	1.5331	1.4846
C12-C13	1.5105	1.5279
C13-C14	1.5024	1.4958
C15-C16	1.5453	1.5093
C16-C17	1.5489	1.5447
C18-C19	1.5637	1.5362
C19-C20	1.5305	1.5472
C20-C21	1.4732	1.5152
C20-C22	1.5060	1.5089
C23-C24	1.4763	1.5467
C24-C25	1.5331	1.5655
C1-O1	1.2137	1.2534
C1-O2	1.2268	1.2409
C4-O3	1.2829	1.2514
C6-O4	1.4812	1.4288
C7-O5	1.2488	1.2164
C9-O6	1.2851	1.2515
C15-O7	1.2394	1.2179
C17-O8	1.4661	1.4136
C18-O9	1.2615	1.2145
C23-O10	1.2299	1.2159
C25-O11	1.4316	1.5011
Ce-O1	-	2.2786
C2-N1	1.4939	1.5766
C4-N1	1.4110	1.4073
C5-N2	1.4905	1.4679
C7-N2	1.2918	1.3803
C8-N3	1.4378	1.4983
C9-N3	1.3466	1.3504
C10-N4	1.5058	1.4311
C15-N4	1.3556	1.4257

C14-N5	1.4959	1.3507
C16-N6	1.4636	1.4006
C18-N6	1.3339	1.3787
C19-N7	1.4333	1.4624
C23-N7	1.3767	1.3376
C24-N8	1.4605	1.447
Bond angle (°)		
O1C1O2	124.456	120.235
O1C1C2	117.683	113.501
C1C2C3	113.682	107.495
C3C2N1	107.722	103.667
C2N1C4	132.027	122.232
N1C4O3	127.606	122.555
N1C4C5	107.324	117.363
C4C5C6	111.780	115.875
C4C5N2	105.496	109.501
C5C6O4	115.894	114.273
C6C5N2	110.727	110.906
C5N2C7	118.741	135.384
C7C8N3	108.519	104.413
O5C7N2	122.023	125.726
O5C7C8	123.462	118.83
C8N3C9	126.798	117.334
N3C9C10	105.312	122.729
N3C9O6	128.242	117.42
C9C10C11	111.252	112.012
C9C10N4	103.454	111.087
C10C11C12	109.231	114.513
C11C12C13	112.098	110.886
C12C13C14	113.080	114.214
C13C14N5	115.570	113.839
C10N4C15	115.276	116.655
N4C15O7	129.172	128.845
O7C15C16	121.065	116.099
N4C15C16	109.382	114.522
C15C16C17	114.826	114.633
C16C17O8	114.41	106.368
C15C16N6	115.987	108.85
N6C18O9	123.116	124.192
N6C18C19	122.400	112.416
C18C19C20	112.241	114.57
C18C19N7	111.822	106.277
C19C20C21	111.871	109.384

C19C20C22	111.211	113.316
C22C20C21	110.864	109.316
C19N7C23	123.953	117.886
N7C23O10	130.428	123.659
N7C23C24	110.291	112.923
C23C24N8	110.610	110.153
C23C24C25	119.489	110.078
N8C24C25	103.383	116.157
C24C25O11	104.396	103.407
Twisting angles (°)		
O1C1C2C3	-89.936	117.426
O2C1C2C3	96.667	-70.734
C1C2N1C4	-118.217	-156.834
C3C2N1C4	119.756	87.806
C2N1C4C5	-8.475	-172.789
N1C4C5C6	-72.483	-34.445
C4C5N2C7	-170.290	-161.944
C6C5N2C7	68.606	68.941
C5N2C7C8	-172.788	-173.615
N2C7C8N3	-125.947	-90.322
C7C8N3C9	165.072	-81.191
C8N3C9C10	173.022	132.697
N3C9C10N4	110.369	72.717
N3C9C10C11	-122.800	-163.468
C9C10C11C12	72.306	67.193
C10C11C12C13	89.940	-168.541
C11C12C13C14	148.389	58.083
C12C13C14N5	-116.935	-128.076
C9C10N4C15	-74.515	-75.787
C11C10N4C15	162.589	159.414
C10N4C15C16	-159.939	-162.423
N4C15C16C17	-167.732	129.092
C15C16N6C18	59.182	56.14
C17C16N6C18	-74.582	-71.626
C16N6C18C19	162.834	170.334
N6C18C19C20	65.988	19.977
N6C18C19N7	-55.572	-89.443
C18C19N7C23	-118.848	-71.028
N7C23C24C25	-53.720	-51.018
C23C24C25O11	-100.622	-172.441

Table S2 The information of the primers used in qRT-PCR performances

Gene	Gene locus	Forward primer [F(5'-3')]<br>Reverse primer [R(5'-3')]
ATFLA11	AT5G03170	F: AGAAAGGCGGCTCTGTTTCA<br>R: TCCCAAACCCGAATCCAGTC
