# Supplementary material for: Arabinogalactan Proteins Are the Possible Extracellular Molecules for Binding Exogenous Cerium(III) in the Acidic Environment Outside Plant Cells
Source: Front Plant Sci. 2019 Feb 20;10:153. doi: 10.3389/fpls.2019.00153 (PMC6391350; doi:10.3389/fpls.2019.00153)
Supplement: Supplementary file 2 [file Data_Sheet_2.pdf]

SUPPLEMENTARY

Arabinogalactan Proteins are the Possible Extracellular Molecules for Binding Exogenous Cerium(III) in the Acidic Environment outside Plant Cells

Qing Yang [1†], Lihong Wang [2†], Jingfang He [1], Haiyan Wei [1], Zhenbiao Yang [3], Xiaohua Huang [1, *]

[1] *National and Local Joint Engineering Research Center of Biomedical Functional Materials, Jiangsu Collaborative Innovation Center of Biomedical Functional Materials, School of Chemistry and Materials Science, Nanjing Normal University, Nanjing 210046, China*

[2] *State Key Laboratory of Food Science and Technology, Jiangnan University, Wuxi 214122, China*

[3] *Center for Plant Cell Biology, Institute for Integrative Genome Biology, University of California, Riverside, CA 92521, USA*

[†] These authors contributed equally to this paper.

* Corresponding author. Email:

huangxiaohuanjnu@yahoo.com (X. Huang)

Building 3D model of ATFLA11 molecule and calculating the surface charge distribution of ATFLA11

We obtained the amino sequence of ATFLA11 (AT5G03170.1) from The *Arabidopsis* Information Resource (TAIR). The structures of the homologous proteins were searched by using Basic Local Alignment Search Tool (BLAST), and finally, four proteins (PDB codes: 1W7D, 1NYO, 1X3B and 2QAE) were searched to be the templates. By using Discovery Studios 2.5 (DS 2.5), we built the 3D model of ATFLA11 molecule (Figure S1A), then the model was successively optimized based on CHARMm force field. Water was selected as the solvent. The pH value and the ion strength was set to be 6.5 and 0.1, respectively. The reliability of the model was verified by using Ramanchandran Plot (Figure S1B). DS2.5 was also used for calculating the surface electron distribution of ATFLA11 (Figure S2).

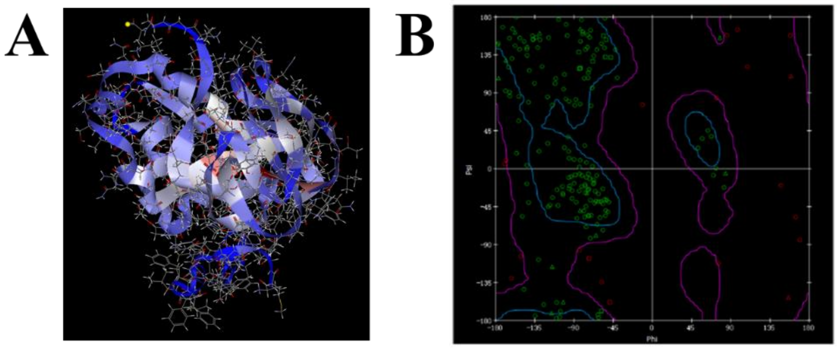

Figure S1 The 3D model of ATFLA11 built by DS2.5 (The yellow atom is the O atom that coordinated to Ce(III) in the result of molecular dynamic simulation) (A) and the result of Ramanchandran Plot (B).

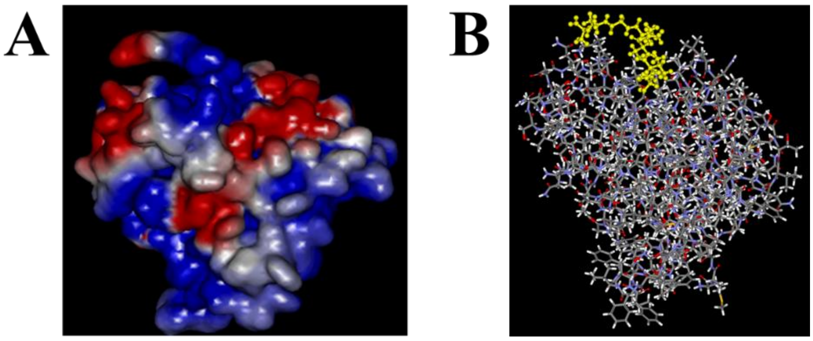

Figure S2 The charge distribution on the surface of ATFLA11 at pH 6.0 (A) and the yellow part in the 3D model of ATFLA11 was chosen to be shown in Figure 8 (B). The positively and negatively charged area is represented by the blue and red area, respectively.

Determination of the relative expression level of gene encoding AtFLA11

The total RNA in *Arabidopsis* leaf cells treated with Ce(III) at different concentrations was isolated using the Total RNA Purification Kit (Sangon Biotech, No. SK8661) according to the instruction. Quantitative real-time reverse transcription-polymerase chain reaction (qRT-PCR) was then performed with AMV first strand cDNA synthesis kit according to the instruction. Three replicates were performed for each treatment group using ABI SybrGreen PCR Master Mix(2X) and ABI Stepone plus qRT-PCR instrument. The primers were designed using Primer Premier 5.0, and the primers were listed in Table S2. Each treatment group had three replicates. The results are shown in Figure S3.

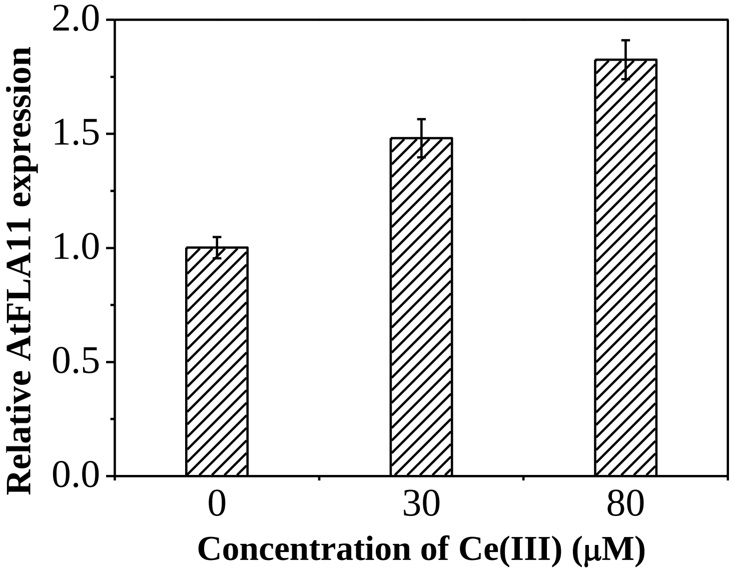

Figure S3 The qPT-PCR results of the expression level of gene encoding AtFLA11 in *Arabidopsis* leaf cells in the presence of Ce(III) at 0, 30 and 80 μM. The measurement was carried out 12 hours after the treatments.
